# Diagnostic accuracy of ELISA for detecting serum Midkine in cancer patients

**DOI:** 10.1371/journal.pone.0180511

**Published:** 2017-07-07

**Authors:** Xuan Jing, Xiangrong Cui, Hongping Liang, Chonghua Hao, Chongyang Han

**Affiliations:** 1 Department of Clinical Laboratory, Shanxi Provincial People's Hospital, Affiliate of Shanxi Medical University, Taiyuan, P.R. China; 2 Reproductive Medicine Center, Children's Hospital of Shanxi and Women Health Center of Shanxi, Affiliate of Shanxi Medical University, Taiyuan, Shanxi, P.R. China; 3 Department of Nephrology, Shanxi Provincial People's Hospital, Affiliate of Shanxi Medical University, Taiyuan, P.R. China; Hokkaido Daigaku, JAPAN

## Abstract

Midkine (MK) has been reported as the potential novel diagnostic biomarker for cancer in several studies, but their results were controversial. Therefore, we performed a diagnostic meta-analysis to assess the diagnostic value of serum MK in cancer patients. A systematic electronic and manual search was performed for relevant literatures through several databases up to June 1, 2017. The quality of the studies included in the meta-analysis was assessed using the Quality Assessment of Diagnostic Accuracy Studies (QUADAS-2) tool. All analyses were conducted using stata12.0 software. Ten studies collectively included 1119 cancer patients and 1441 controls met the eligible criteria. The summary estimates were: sensitivity 0.78 (95% CI = 0.68–0.85), specificity 0.83 (95% CI = 0.72–0.90), positive likelihood ratio 4,54 (95% CI = 2.64–7.80), negative likelihood 0.27 (95% CI = 0.18–0.40), diagnostic odds ratio 16.79 (95% CI = 7.17–39.33), and area under the curve 0.87 (95% CI = 0.84–0.89). Publication bias was suggested by Deeks’ funnel plot asymmetry test (*P* = 0.92). According to our results, serum MK has greater diagnostic value in diagnosing cancer, however, more reliable studies in larger cohort should be conducted to evaluate the diagnostic accuracy of serum MK.

## Introduction

Cancer is among the leading cause of morbidity and mortality across the globe, accounting for 17.5 million cancer cases worldwide and 8.7 million deaths in 2015, making it a global health problem with a heavy burden on the society[1–3]. The increasing morbidity and mortality from cancer might attribute to earlier diagnosis and treatment[4, 5]. Most of the cancer patients could have a high chance of cure if diagnosed early and treated adequately. Growing evidences suggested that the five-year survival rate could be improved significantly with diagnosis in the early stage of cancer such as gastric cancer, lung cancer, and renal cancer[6–8]. Unfortunately, the majority of cancer patients are diagnosed in advanced stages where early metastasis to lymph nodes, adjacent tissue or organs occurs, following with a reduced overall five-year survival rate. Therefore, it is urgent to find accurate and efficient methods for cancer diagnosis at its early stage to improve the survival status for cancer patients and simultaneously reduce the cancer burden.

Biopsy is currently considered the golden standard for diagnosing solid tumor, often combined with ultrasound and imageological examination[9]. However, the invasive nature of biopsy and low sensitivity of ultrasound and imageological examination restrict their application in the early diagnosis of tumor[10, 11]. Therefore, exploring more reliable non-invasive detection of new or recurrent tumor is the need of the hour.

Midkine (MK), a heparin-binding growth factor, was a highly expressed factor during the early differentiation stage in embryonal carcinoma and was weak or undetectable in normal adult tissues[12–14]. Mounting evidence has indicated that MK plays an essential role in various biological activities in malignant tumors such as proliferation, migration, antiapoptosis, angiogenesis, invasion, and metastasis[15]. Increased MK mRNA and protein expressions have been reported in many early stage human cancers tissue, such as breast cancer, gastrointestinal cancer, and lung cancer[16–19]. Due to the secretory protein properties, the detection of MK in serum or plasma could serve as a diagnostic marker and allow the early detection of malignancy. Nowadays, the diagnostic value of MK for the early detection of cancer has been successfully detected in various tumors, such as duodenal, colon, pancreatic, lung, esophageal, tumors and hepatocellular carcinoma[19–21].

Despite many studies have demonstrated the potential of serum or plasma MK as a novel marker for tumor diagnosis, the previous studies have been limited by relatively small sample size recruited in the individual studies, and still no comprehensive conclusion have been reached for the diagnostic value of MK in detection cancers. Therefore, the aim of the present study was to review and assess the overall diagnostic test accuracy of serum MK for cancer diagnosis.

## Results

### Included studies

**Fig 1:**
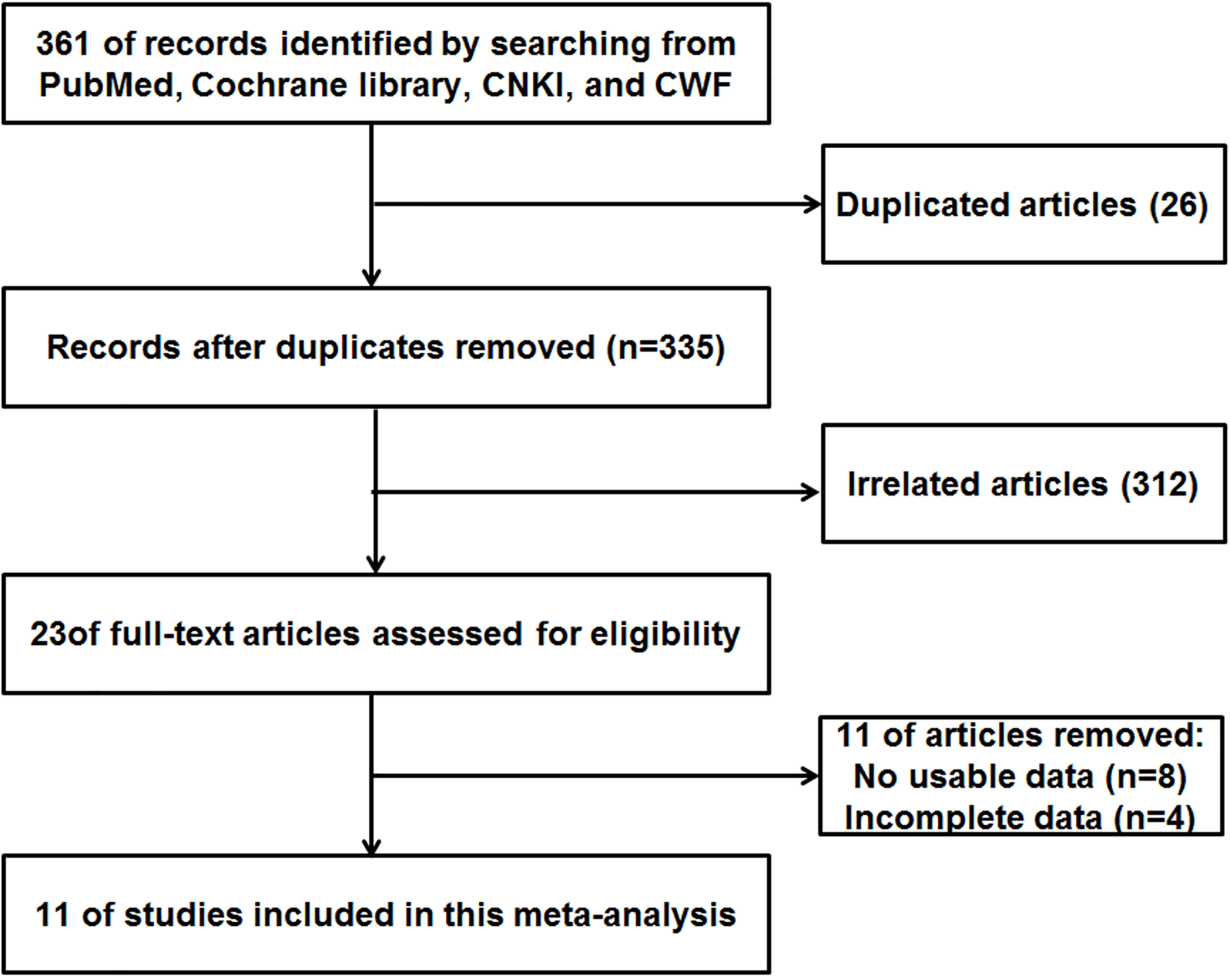
The flow diagram of this meta-analysis.

A detailed flowchart of our literature research is presented in Fig 1. The initial search returned a total of 361 articles involving MK and cancer, and 26 of them were excluded due to duplications. The remaining 335 articles were subjected to the next stage of evaluation. Among the remaining 312 excluded articles, 178 were reviews, letters, meta-analyses or not related to the research topic, 36 were not from serum, 87 were not related to cancer diagnose and 11 articles did not contain sufficient and useful data. Finally, 11 articles were included in this meta-analysis[21–30].

### Study characteristics and quality assessment

The main characteristics of the included studies were summarized and listed in Table 1 by order of publication year, ranging from 2003 to 2017. A total of 2826 subjects (1128 Cancer patients and 1574 controls) from 11 studies were included in the meta-analysis. Among the 11 studies, 4 were conducted in China, 2 in Japan, 1 in Germany, 1 in Poland, 1in Egypt, 1 in Australia, 1 in Turkey. In addition, different types of cancer were recorded including esophageal squamous cell carcinoma (n = 1), pediatric embryonal tumor (n = 1), colorectal cancer (n = 1), hepatocellular carcinoma (n = 3), thyroid cancer (n = 1), non-small cell lung cancer (n = 2), mesothelioma (n = 1), head and neck squamous cell carcinoma (n = 1). All studies used the ELISA to measure the levels MK in serum or plasma. The QUADAS-2 scores of the included studies were shown in Table 1. Overall, the majority of studies presented a moderate-high quality.

**Table 1:** Characteristic of the included studies.

Study ID	Country	Ethnicity	Cancer	Sample size			Cut-off	Diagnostic power				QUADAS
				Control	Case	Total		TP	FP	FN	TN	5
Hideaki Shimada, 2003	Japan	Asian	ESCC	151	93	244	300pg/ml	57	6	36	145	4
Susanne Lucas, 2009	Germany	Caucasian	PET	152	29	181	0.176ng/ml	25	52	4	100	4
Malgorzata, 2013	Poland	Caucasian	CRC	156	105	261	406ng/L	87	50	18	106	5
Wenwei Zhu, 2013	China	Asian	HCC	505	302	807	0.654ng/ml	262	81	40	424	4
Zhaowei Meng, 2015	China	Asian	DTC	75	70	145	323.48pg/ml	53	15	17	60	5
Karim Y.A. Shaheen, 2015	Egypt	Caucasian	HCC	30	40	70	NA	40	1	0	29	3
Changming Sun, 2015	China	Asian	NSCLC	52	52	104	NA	42	3	10	49	3
Roslyn Vongsuvanh, 2016	Australia	Caucasian	HCC	172	86	256	0.44ng/ml	61	65	25	107	5
Xin Xia, 2016	China	Asian	NSCLC	118	153	271	400pg/ml	109	14	44	104	5
Taku Yamashita, 2016	Japan	Asian	HNSCC	116	103	345	482pg/ml	59	17	44	99	4
Gunulu Ak, 2017	Turkey	Caucasian	Mesothelioma	47	95	142	421pg/ml	58	23	37	24	5

### Diagnostic accuracy and threshold analysis

**Fig 2:**
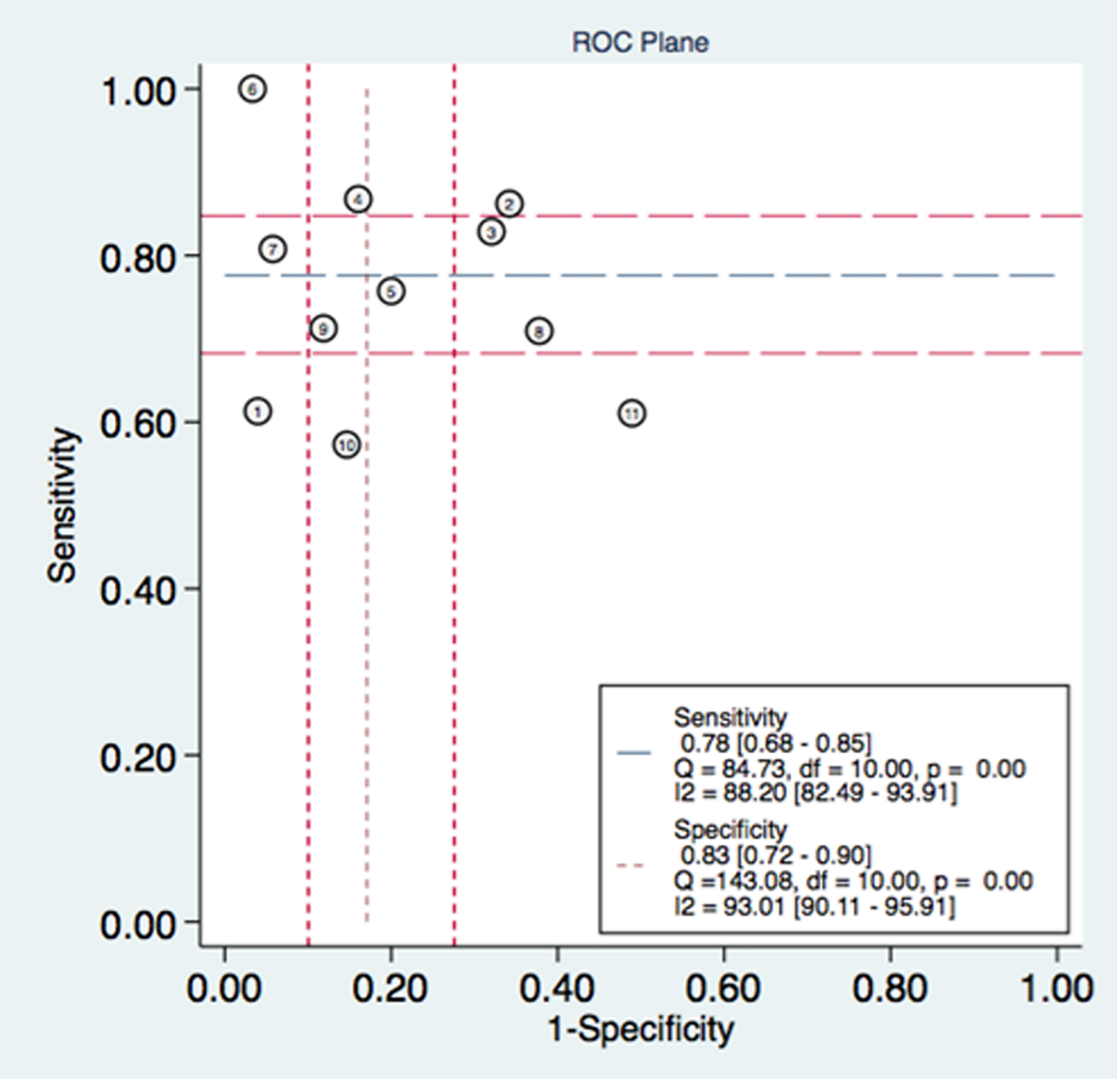
ROC plane.

**Fig 3:**
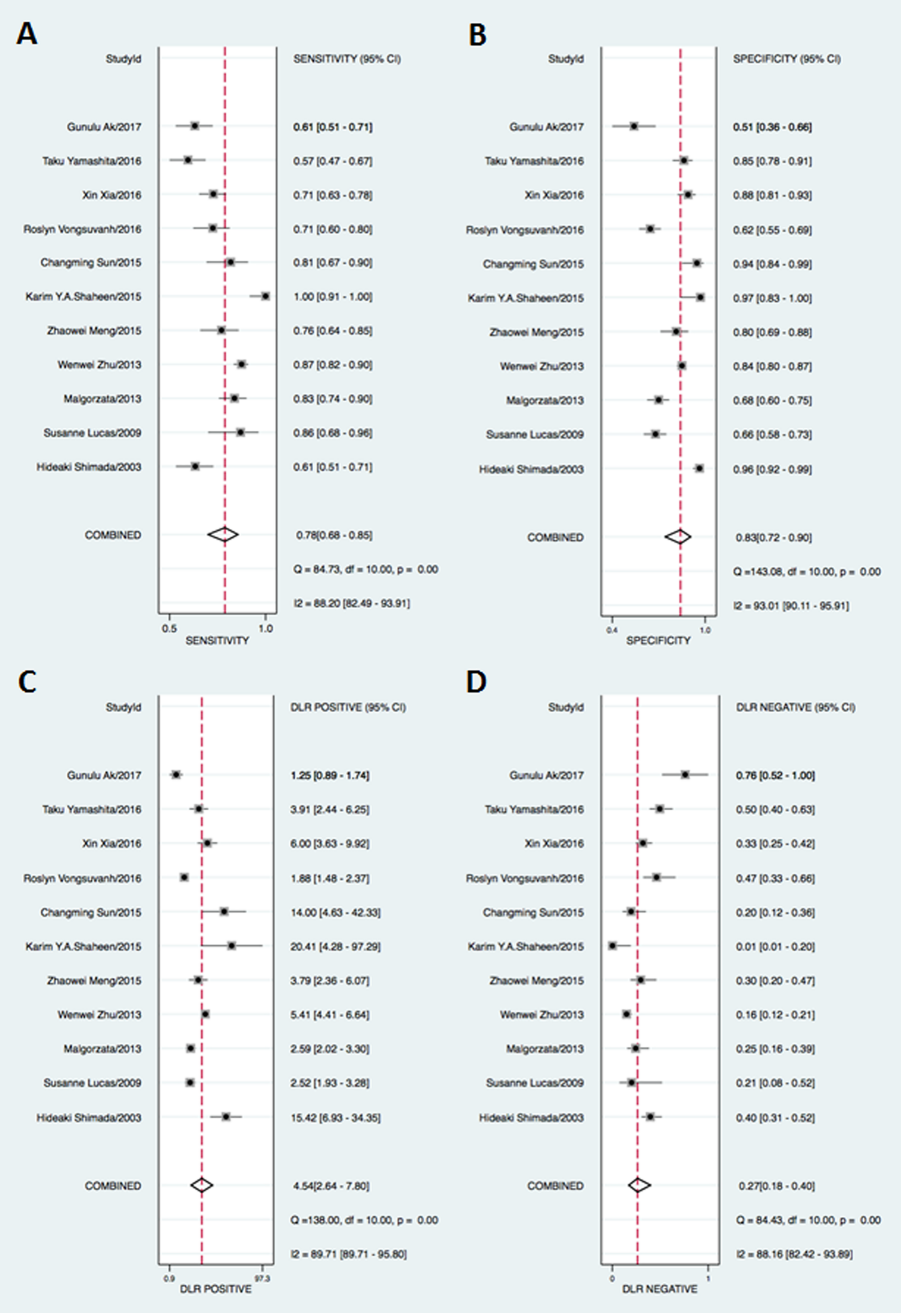
Forest plot of sensitivity(A), specificity(B), positive likelihood(C),negative likelihood(D) with their 95%CIs.

**Fig 4:**
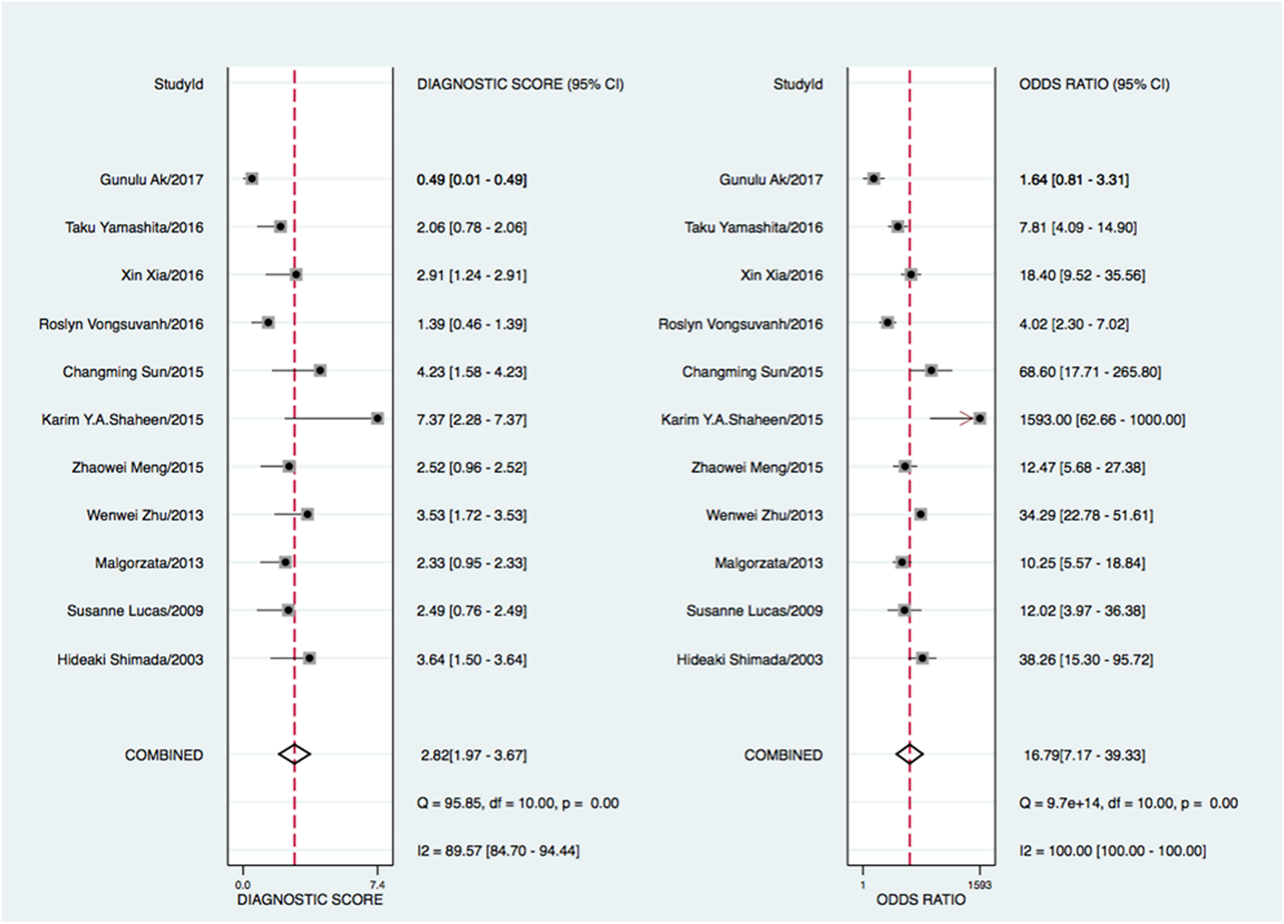
Forest plot of diagnostic odds ratio estimates and 95% CI.

**Fig 5:**
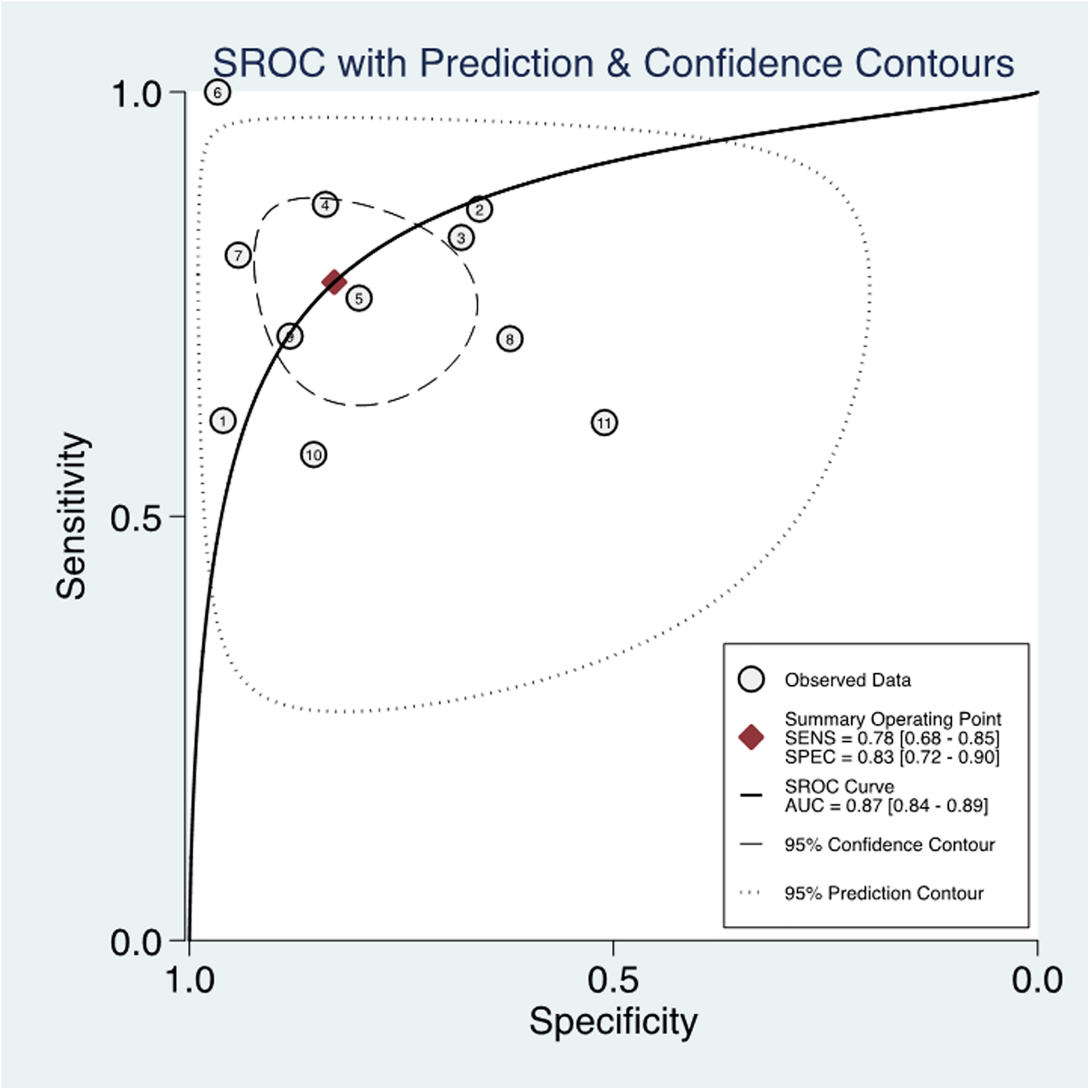
Summary receiver operating characteristic graph of included studies.

In this meta-analysis, the threshold effect, an inverse correlation between the sensitivity and specificity, was conducted to explore whether it was existed in the study, which was shown in an ROC plane (Fig 2). The shape of ROC curve showed a nontypical shoulder-arm appearance and the spearmen correlation coefficient value of sensitivity and 1-specificity was -0.1455 with a *P* value was 0.6696 suggesting that there was no heterogeneity from the threshold effect. The forest plot of sensitivity and specificity for MK assays was shown in Fig 3. Heterogeneity in sensitivity and specificity was detected in 11 studies (I^2^ = 88.20 and 93.01, respectively), suggesting that significant heterogeneity was existed in sensitivity and specificity from non-threshold effect. Therefore, the random effects model was employed in this study. The pooled sensitivity, specificity, PLR, NLR, and DOR with their 95% confidence intervals (CIs) were 0.78 (95% CI: 0.68–0.85, Fig 3A), 0.83 (95% CI: 0.72–0.90, Fig 3B), 4.54 (95% CI: 2.64–7.80 Fig 3C), 0.27 (95% CI: 0.18–0.40, Fig 3D), and 16.79 (95% CI: 7.17–39.33, Fig 4), respectively. The SROC curve for the 11 included studies is shown in Fig 5. The AUC of MK tests was 0.87 (95% CI = 0.84–0.89), thereby implying a relatively high diagnostic accuracy.

### Meta-regression and sensitivity analyses

**Fig 6:**
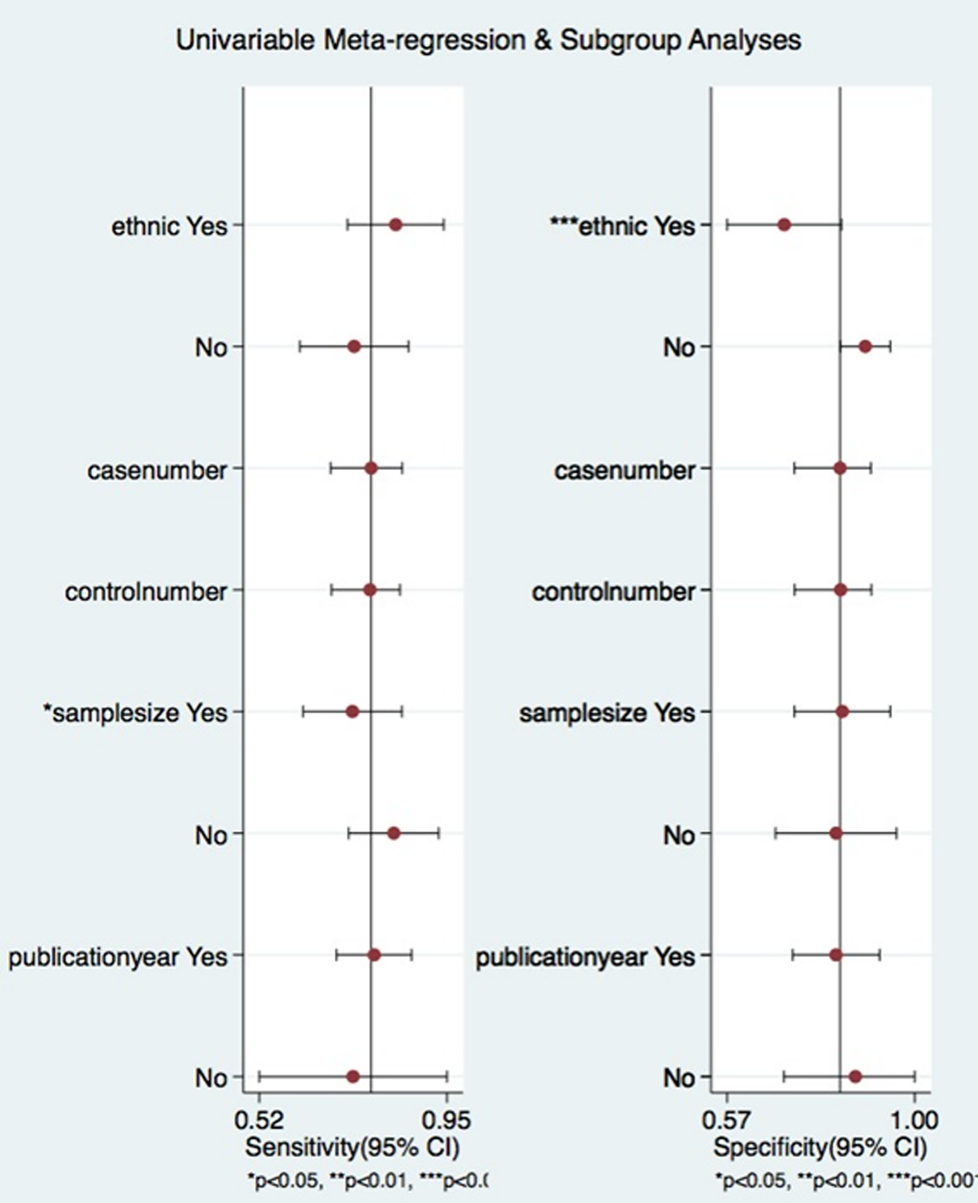
Meta-regression to explore the heterogeneity between studies.

Meta-regression analysis was performed to explore the potential source of heterogeneity in sensitivity and specificity base on the covariates including ethnicity, simple size, number of case and control, year of publication. The results in Fig 6 showed that sample size (*P*<0.01) had an effect on sensitivity, while the ethnicity (*P*<0.01) contributed to interstudy heterogeneity for specificity.

**Fig 7:**
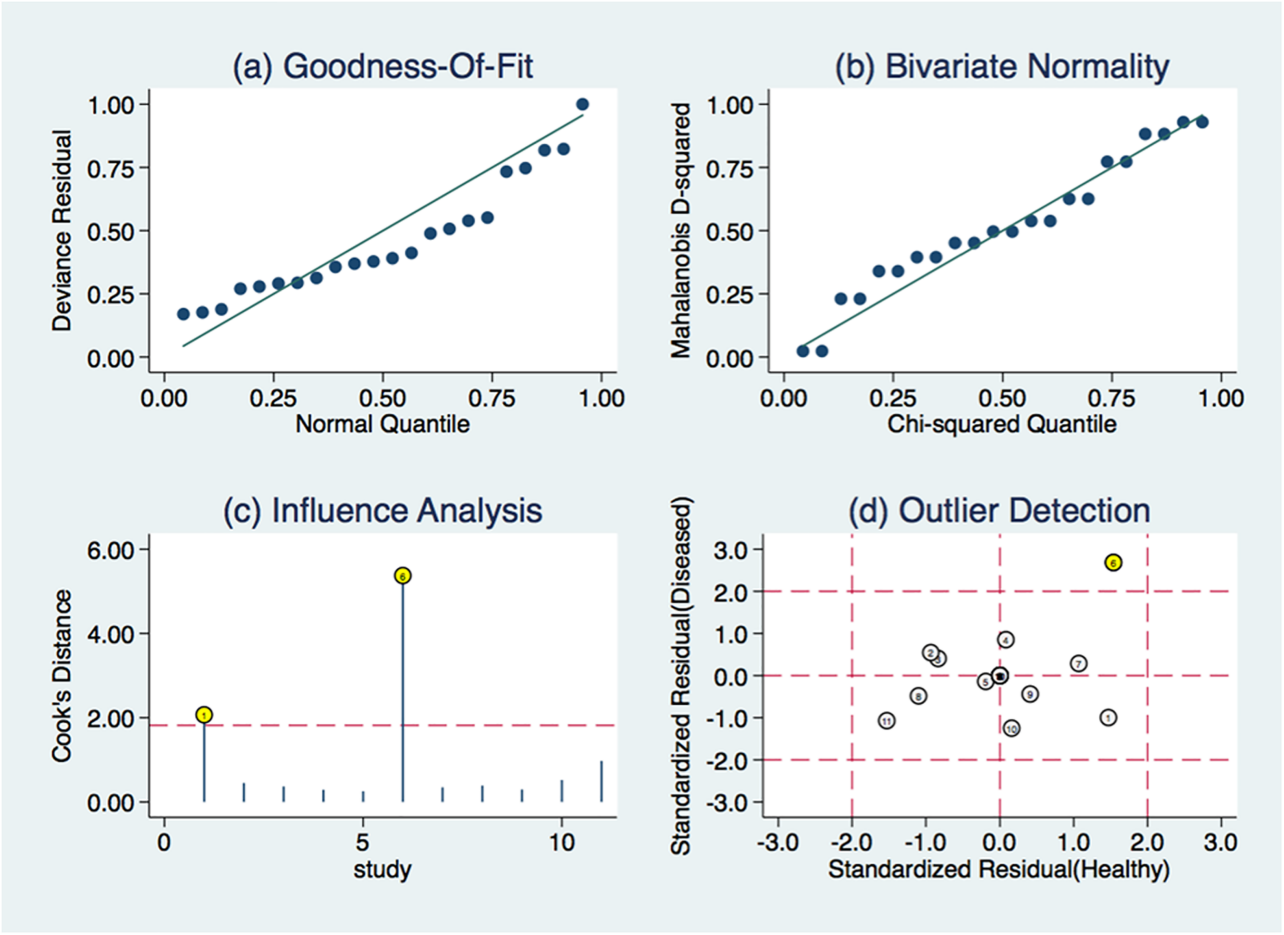
Graphical depiction of residual-based goodness-of-fit(A), bivariate normality(B), influence(C) and outlier detection(D).

Additionally, sensitivity analyses was conducted and 2 outlier was found by influence analysis (Fig 7C) and outlier detection (Fig 7D). After the one outlier excluded from the test, sensitivity (from 0.78 to 0.74) and specificity (from 0.83 to 0.81), AUC (from 0.87 to 0.83) showed minimal changes, indicating the outlier did not have a substantial effect on the overall analysis. Combined with goodness of fit **(**Fig 7A**)** and bivariate normality analyses (Fig 7B), we confirmed that the random-effect bivariate model was moderately robust for this meta-analysis.

### Publication bias

**Fig 8:**
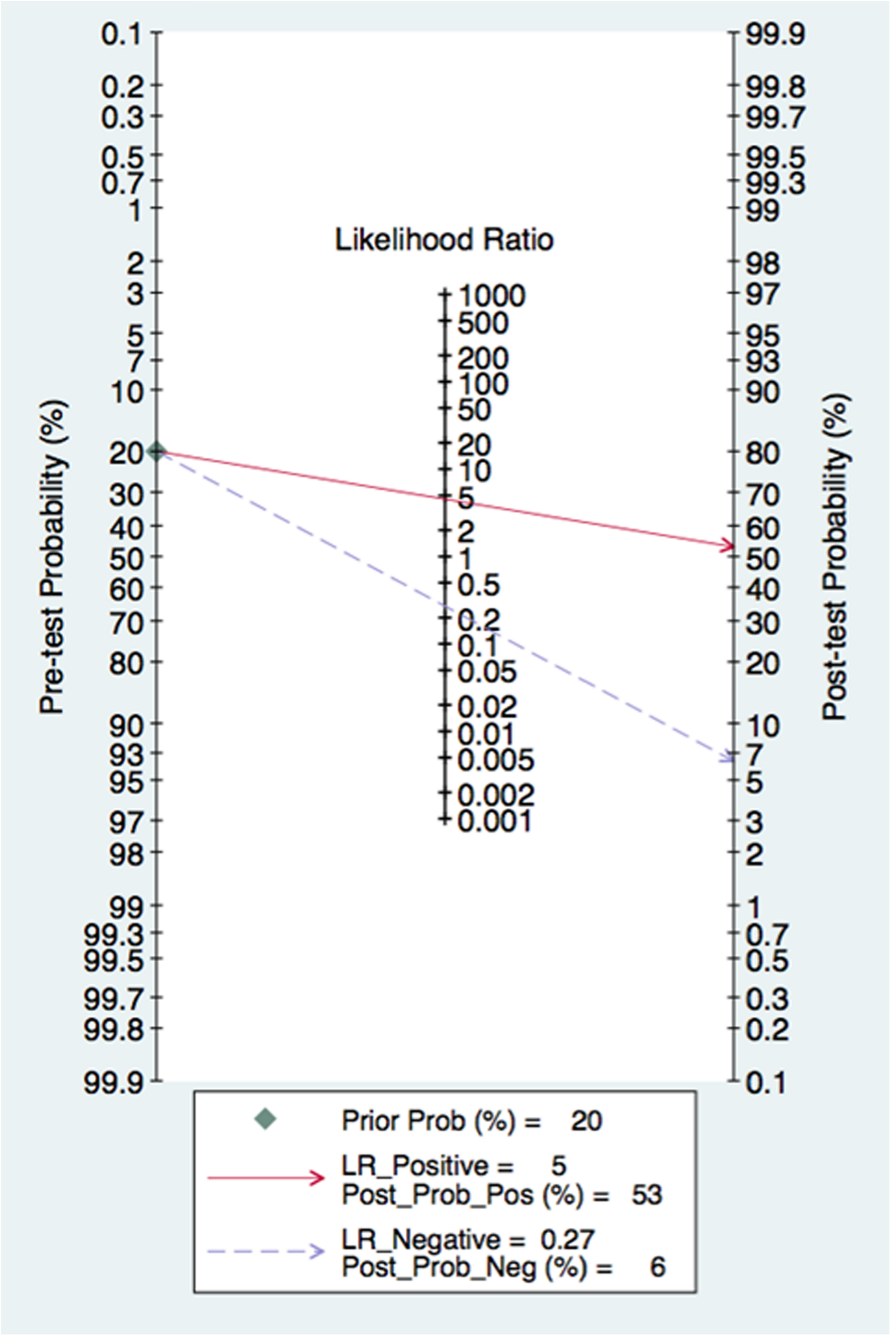
Fagan’s nomogram for estimating post-test possibilities.

**Fig 9:**
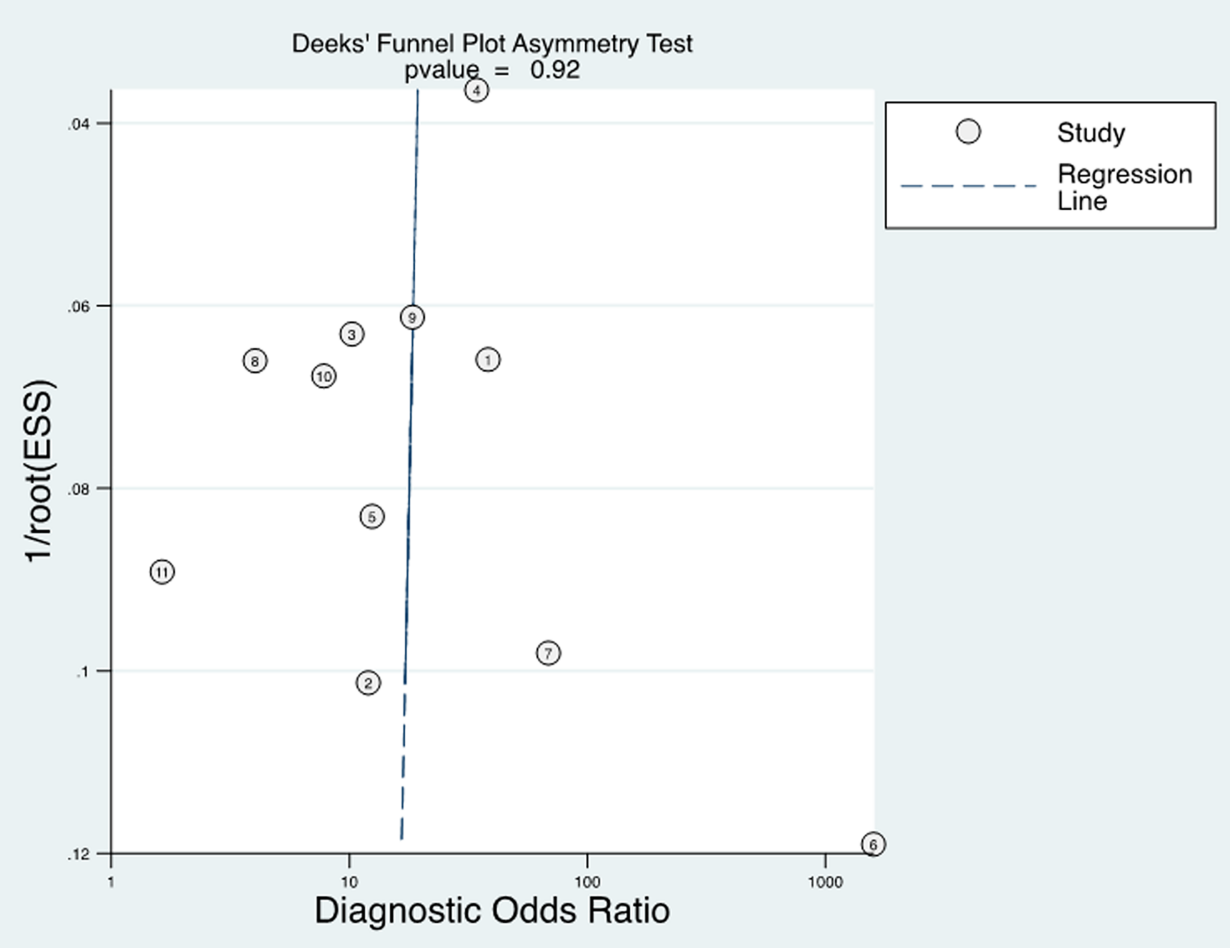
Deeks’ funnel plot asymmetry test for publication bias.

Fagan’s Nomogram for likelihood ratio in Fig 8 showed that when a pre-test probability of 20% was specified, the post-test probability positivity would raise to 53% with a PLR of 5, and the post-test probability negativity would decreased to 6% with a NLR was 0.27. These results suggest a moderate value for MK in the diagnosis of cancer in serum. In addition, we conducted Deeks’ test to assess the publication bias and found no significant publication bias in our study with *P* value 0.92 (Fig 9).

## Discussion

Cancer continues to be a major public health challenge and an important cause of mortality and morbidity worldwide[31]. Early diagnosis and treatment are the key point for the clinical course and the outcome patients with cancer[32, 33]. Cancer-specific variations in secreted proteins in the blood are promising noninvasive biomarkers for identifying and monitoring patients with cancer. MK, as one of the most extensively studied aberrant secreted proteins, was discovered to be increased in the early stages of numerous tumors[16]. In 1999, Konishi N *et al* revealed that MK is highly expressed in precancerous lesions and the early stage of prostate carcinoma development[34]. Furthermore, abnormal high expression of MK could promote cell proliferation, angiogenesis, invasion, migration and metastasis[35]. Meanwhile, as a feasible and non-invasive alternative, MK was promising to be a biomarker for cancer. However, the diagnosis accuracy of serum MK has been reported to be variable. Hence, we conducted a comprehensive meta-analysis to evaluate the diagnostic accuracy of MK for cancers.

In the present meta-analysis, we performed a diagnostic meta-analysis to assess the accuracy of serum MK as a biomarker for cancer. The pooled sensitivity and specificity of serum MK were 0.78 (95% CI = 0.68–0.85) and 0.83 (95% CI = 0.72–0.90) respectively, indicating that 78% of cancer patients had high serum MK levels and that 83% of the non-cancer patients had low serum MK levels. The pooled DOR of 16.79 (95% CI = 7.17–39.33) suggested that the overall accuracy of serum MK for the diagnosis of cancer is credible. The likelihood ratio, including PLR and NLR, could also reflect the diagnostic accuracy. When PLR>10 or NLR<0.1, the likelihood of diagnosis or exclusion of a disease increased remarkably. Nevertheless, in our meta-analysis, a pooled PLR of 4.54 (95% CI = 2.64–7.80) and NLR 0.27 (95% CI = 0.18–0.40) suggested that MK may not be powerful enough to confirm or exclude the potential patient with cancers. However, an AUC of 0.87 (95% CI = 0.84–0.89) means a high ability for cancer detection. Therefore, serum MK was an effective biomarker for cancer diagnosis.

Heterogeneity is a potential problem that can influence the incorporation effect and the interpretation of the results[36]. The shape of ROC curve and the spearmen analysis suggested that heterogeneity could not be explained by a threshold effect. Meta-regression analysis was performed to explore the potential source of heterogeneity in sensitivity and specificity base on the covariates including ethnicity, simple size, number of case and control, year of publication. The results indicated that sample size had an effect on sensitivity, while the ethnicity contributed to interstudy heterogeneity for specificity. Due to there were insufficient eligible studies to fully elucide the source of the heterogeneity, the possible reason for the heterogeneity need to be investigated in future studies.

The current study has several limitations. First, despite extensive literature search were performed, the limited number of included studies and cancer types may restrict our ability to evaluate the accuracy of serum MK. Second, we could not determine the ideal cut-off value for serum MK test, due to inconsistent cut-off values were adopted in each study. Third, the majority of these studies are retrospective analyses on historical cohorts, which may limit the conclusion due to selection bias. Fourth, only articles published in English or Chinese were enrolled in our meta-analysis, and studies published in other languages were not included, which may cause inevitable bias.

Despite these limitations, the present evidence indicates that serum MK is potential to be a diagnosis biomarker for cancer, because this non-invasive method has good overall diagnostic performance. However, large-scale and comprehensive studies must be performed in the future to validate this finding.

## Materials and methods

### Search strategy and selection criteria

This meta-analysis was performed in accordance with the Preferred Reporting Items for Systematic reviews and Meta-Analyses (PRISMA) guidelines[37] Studies were systematically searched from PubMed, Google Scholar, EMBASE, Chinese National Knowledge Infrastructure (CNKI), Wan Fang Database up to June 1, 2017 with the following MeSH and key words:(”midkine” or “MK” or “MDK”), and “serum” and (“cancer” or “carcinoma” or “tumor neoplasm”) and (“diagnosis” or “ROC curve”) without language restriction or publication date restrictions. In addition, references of all articles in these eligible studies were also read to identify additional relevant literature.

All studies should meet the following criteria: (1) studies appraising MK for cancer diagnosis; (2) the levels of MK in serum or plasma were determined; (3) sufficient information were provided or can be used to calculate the sensitivity and specificity (including true positive-TP, false positive-FP, false negative-FN and true negative-TN). While the exclusion criteria were list as follows: (1) duplications or overlapping studies; (2) studies without adequate data to construct the 2×2 table; (3) reviews, letters, case report and conference abstracts.

### Data extraction and quality assessment

Data extraction from eligible studies was performed independently by 2 investigators, and disagreements were resolved by deliberation with a third investigator. From each study, the following study characteristics were extracted: (1) basic information of articles (the first author, year of publication, country of publication); (2) research object’s general features (ethnicity, sample size, mean age of subjects, type of cancer); (3) data used for our final meta-analysis (detection method, cut-off value, sensitivity, specificity, TP, FP, FN, and TN); (4) other research features (sample source, study design, etc).

The Quality Assessment of Diagnostic Accuracy Studies-2 (QUADAS-2) instrument, a quality assessment tool, was used to assess the methodological quality of individual studies by the same two independent investigators[38]. The QUADAS-2 tool consisted of four domains: Patient selection, index test, reference standard, and flow and timing. Each of the assessment has seven questions, which should be answered with “yes”, “no”, or “unclear”. An answer of “yes” gets 1 score, while others get 0, the highest score is seven[39].

### Statistical analysis

All the analyses of diagnostic accuracy were performed using STATA 12.0 software. The bivariate meta-analysis model was used to calculate the pooled results of sensitivity, specificity, positive and negative likelihood ratios (PLR and NLR), diagnostic odds ratio (DOR) with their corresponding 95% confidence intervals (CIs)[40, 41]. The summary receiver operator characteristic (SROC) curve and the area under the SROC curve (AUC) were generated to evaluate the accuracy of cancer diagnosis. An AUC of 1.0 indicates perfect diagnostic accuracy whereas an AUC of 0.5 indicates poor diagnostic accuracy[42]. Additionally, the Spearman correlation coefficient was used to evaluate the diagnostic threshold effects, and *P*<0.05 indicates significant heterogeneity. The statistical heterogeneity from non-threshold was assessed by the Q value and I^2^ statistic. A probability value of *P*<0.05 and I^2^≥50% indicated the existence of significant heterogeneity among individual studies[43]. Furthermore, meta-regression and subgroup analyses were conducted to explore the potential sources of heterogeneity. The potential publication bias was estimated by Deeks’ funnel plot and a probability value of *P*<0.05 indicates significant publication bias[41]. Finally, the Fagan’s nomogram was graphed to estimate the post-test probabilities.

## Supporting information

S1 Table PRISMA checklist. PRISMA checklist identifying how and where each element of the PRISMA process has been addressed in this paper.
(PDF)
